# Caregiver’s perspectives on facilitators and barriers of active participation in cerebral palsy rehabilitation in North West Nigeria: a qualitative study

**DOI:** 10.1186/s12913-020-05487-w

**Published:** 2020-07-06

**Authors:** Auwal Abdullahi, Auwal Isah

**Affiliations:** 1 Department of Physiotherapy, Bayero University Kano, PMB 3011, Gwarzo road, Kano, Nigeria; 2 Department of Physiotherapy, Aminu Kano Teaching Hospital, Kano, Nigeria

**Keywords:** Cerebral palsy, Rehabilitation, Caregivers, Motivation, Empathy

## Abstract

**Background:**

Cerebral Palsy (CP) refers to the permanent disorders involving postural and movement control as a result of injury to the developing brain. As a result of impairment in postural and movement control, children with CP usually have problems in carrying out activities of daily living (ADL). This makes them dependent on help from their caregivers. Thus, for effective rehabilitation of children with CP, active participation of their caregivers is important. This study seeks to explore the facilitators and barriers of active participation of caregivers in the rehabilitation of children with CP in Kano, Nigeria.

**Methods:**

The study design used was qualitative in-depth interview. The participants were caregivers of children with CP at Hasiya Bayero Paediatric Specialists Hospital, Kano. The caregivers were interviewed face-to-face, and their responses were audio-recorded with a tape recorder, supplemented with note taking. The data generated was analyzed using constant comparative analysis.

**Results:**

Forty young caregivers (mean age, 27.17 ± 4.46 years) participated in the study. They expressed encouragement from the therapist managing the child, family support, empathy, improvement in the conditions of other children with CP, cooperation of the child during home programs family support and improvement in the child’s condition as factors that facilitate their active participation in the rehabilitation of the children. However, they mentioned occupation, financial resources and the number of children the caregiver has are the barriers to their active participation in the rehabilitation of the children.

**Conclusions:**

Both the facilitators and barriers of active participation of caregivers in the rehabilitation of children with CP need to be recognized in order to help caregivers reinforce or overcome them respectively. In addition, economically sustainable and accessible rehabilitation services are needed for all children with CP. Similarly, sharing caregiving rehabilitation tasks amongst family members could facilitate caregiver active participation.

## Background

At particular stages in life, the child is supposed to have certain abilities such as keeping the head and neck upright in sitting, crawling, rolling, standing and walking. These are known as motor milestones [1–3]. Following Cerebral Palsy (CP), these abilities may be either absent or come much later in comparison with a child without CP [4]. Rehabilitation, which is the process of helping people to restore lost function or improve functional independence, can help children with CP. Cerebral Palsy is a long-term condition as it results in permanent disorders of movement and posture [5, 6]. Thus, its rehabilitation may require active participation of the therapists and the caregivers. In Nigeria, rehabilitation services by the therapists are often accessed only in secondary and tertiary facilities in the urban centers, with little or no community rehabilitation services [7]. Recently, it has been suggested that, there are opportunities for rehabilitation therapists to play important roles at the primary healthcare level in the country [8]. However, even this may not be able to adequately cater for the rehabilitation needs of children with CP since they may require care 24/7. Therefore, active participation of the caregivers in the rehabilitation of the children may help supplement their rehabilitation needs.

Caregiver participation in the rehabilitation of children with CP pertains to assisting the children with daily care activities such as bathing, mobility, feeding and dressing [9]. When caregivers participate in the outpatient rehabilitation of their children, their participation can provide a more natural environment for healthy development in physical and psychosocial wellbeing of the children [10]. However, systems of caregiving may differ from one place to another. In Kano, Nigeria where CP prevalence stands at 42% of all paediatic neurology cases [11], caregiving is provided by the family members of the patients. This is in contrast to South Africa where there is an organized community caregiving system, in which care is provided by the community organized by civil societies [12]. Similarly, in western countries, caregiving may be supported by the local health community, foster families or various non-governmental organizations [13, 14].

Although caregivers may participate in developing strategies to aid with development of children with CP, elsewhere, it was noted that several challenges such as environmental barriers, inaccessible health facilities and lack of specialists care may be encountered [15, 16]. Additionally, caregiving was previously reported to cause huge mental and emotional stress as it imposes physical, time and financial demands [17–19]. The aim of this study is to therefore explore the barriers and facilitators of active participation of caregivers of children with CP in the rehabilitation of their children in Kano, Nigeria. Understanding these two processes may help with devising strategies to either reinforce or improve them during rehabilitation.

## Methods

### Design

The study design was qualitative using in-depth interviews (grounded theory approach) to explore the barriers to and facilitators of active participation of caregivers in the rehabilitation of children with CP. In-depth interview methodology was chosen because it provides rigorous and deep responses of participants. Rigor in qualitative research assures quality [20]. However, it worth stating that an abstract of the study methods and findings was published earlier [21].

### Participants

The population of this study was caregivers of children with CP attending Hasiya Bayero Specialists Paediatric Hospital in Kano, Nigeria. In this hospital, children with CP receive rehabilitation by the physiotherapists once per week. Purposive sampling technique was used for the selection of the study participants. In this regard, only primary caregivers such as mothers, aunts, uncles, sisters, brothers and grandmothers who are directly involved in taking care of the children were considered for recruitment into the study. Participants were included if they served as caregivers to a child with CP for at least one year.

For the sample size, there are no hard and fast rules for sample size estimation in qualitative research as this type of study approach maintains some degrees of openness [22]. Consequently, the number of participants available was used until theoretical saturation was attained. Theoretical saturation is a situation in which new interviews no longer produce new information or insights [23, 24]. The data collection instruments used were the study demographic information data sheet, a qualitative interview guide, pen and a notebook/pad and a voice recorder. The qualitative interview guide was piloted first on seven caregivers before the commencement of the main study, and it consisted of the following questions [1] what are the things that hinder you from actively participating in the rehabilitation of your child? 2) What are the things that motivate you or make it easy for you to actively participate in the rehabilitation of your child? Additionally, in each of the above cases/ questions, probing technique to elicit more responses or better understand the responses was used. Two independent colleagues who are familiar with qualitative research methodology reviewed the interview guide and offered suggestions before it was finally produced.

Ethical approval was sought from Research Ethics Committee of Kano State Ministry of Health. Participants’ written consent was obtained after detail explanation of the study to them (this was to seek for their consent and to familiarize ourselves with each other). Participants were interviewed face-to-face individually at different times to ensure their responses were not influenced by others. The data was collected at the Department of Physiotherapy, Hasiya Bayero Specialists Paediatric Hospital in Kano, Nigeria with only the caregiver and the child with CP in attendance. According to Tracy, the aforementioned procedures help to achieve quality assurance in qualitative research [20]. All caregivers approached consented to participate. Furthermore, no repeat interviews were conducted because all the respondents responded well and the required responses were obtained.

The authors (AI and AA), both male, conducted the interview. One of them (AA) holds a Masters degree, a certificate in Qualitative Methods, and has undertaken at least 10 qualitative studies previously. Author AI holds a Bachelors degree and prior to participating in the study, received training on the principles and practice of qualitative research. Researchers and interview participants did not know each other prior to the interview. The study participants were interviewed by the interviewer (AA) using the interview guide. The responses of the study participants were audio-recorded using tape recorder and noted by a note taker (AI). The transcripts were not returned to the respondents for member checking.

### Data analysis

The demographic characteristics of the study participants were analyzed using descriptive statistics of frequency, percentage, mean and standard deviation. The responses of the study participants were analyzed using constant comparative analysis, a grounded theory approach [25, 26]. After collecting the data from all the study participants, constant comparative analysis was used to analyze the data which involved re-reading, categorizing, coding and then connecting the codes (themes) using matrix analysis. The themes were generated from the available data manually using matrix analysis and no software such as NVivo was used. However, participants were not used in checking the themes. All the processes of the analysis were carried out by both authors independently. Disputes during the data analysis especially as regards to the generation of the themes were resolved through discussion and consensus between the two authors.

## Results

There were 40 caregivers of children with CP who participated in the study with mean age, 27.17 ± 4.46 years. The details of the demographic characteristics of the study participants including mean age of the children, occupation of their caregivers, levels of education of the caregivers and sex of the caregivers are presented in Table 1. The mean duration of the interview sessions was 38.8 ± 4.98 min (range 30 to 45 min).

**Table 1:** Demographics and Characteristics of the Study Participants (*N* = 40)

Variable		%
Mean age of the caregivers	27.17 ± 4.46 (21–40) years	
Gender (female/male)	40/0	100%/ 0%
Relationship with the child (mother/others)	38/2	95% /5%
Mean time since caregiving started	2.90 ± 1.55 (1–8) years	
Mean age of the children	3.00 ± 1.68 (1–8) years	
Range of number of children	(2–8)	
Range of number of dependents	(2–9)	
Level of education (primary/ secondary /higher institution/ none)	3/ 31/ 6/ 0	7.5%/ 77.5%/ 15%/ 0%
Religion (Islam/ Christianity/ Others)	40/ 0/ 0	100%/ 0%/ 0%
Occupation (housewife/ business/ civil servant/ others)	23/ 14/ 3/ 0	57.5%/ 35%/ 7.5%/ 0%
Marital status (married (currently)/ single)	40/ 0	100%/ 0%
Tribe (Hausa/ Fulani/ Yoruba/ Igbo	36/ 4/ 0/ 0	80%/ 20%/ 0%/ 0%
Gender of the child (female/male)	28/ 12	70%/ 30%

**Fig. 1:**
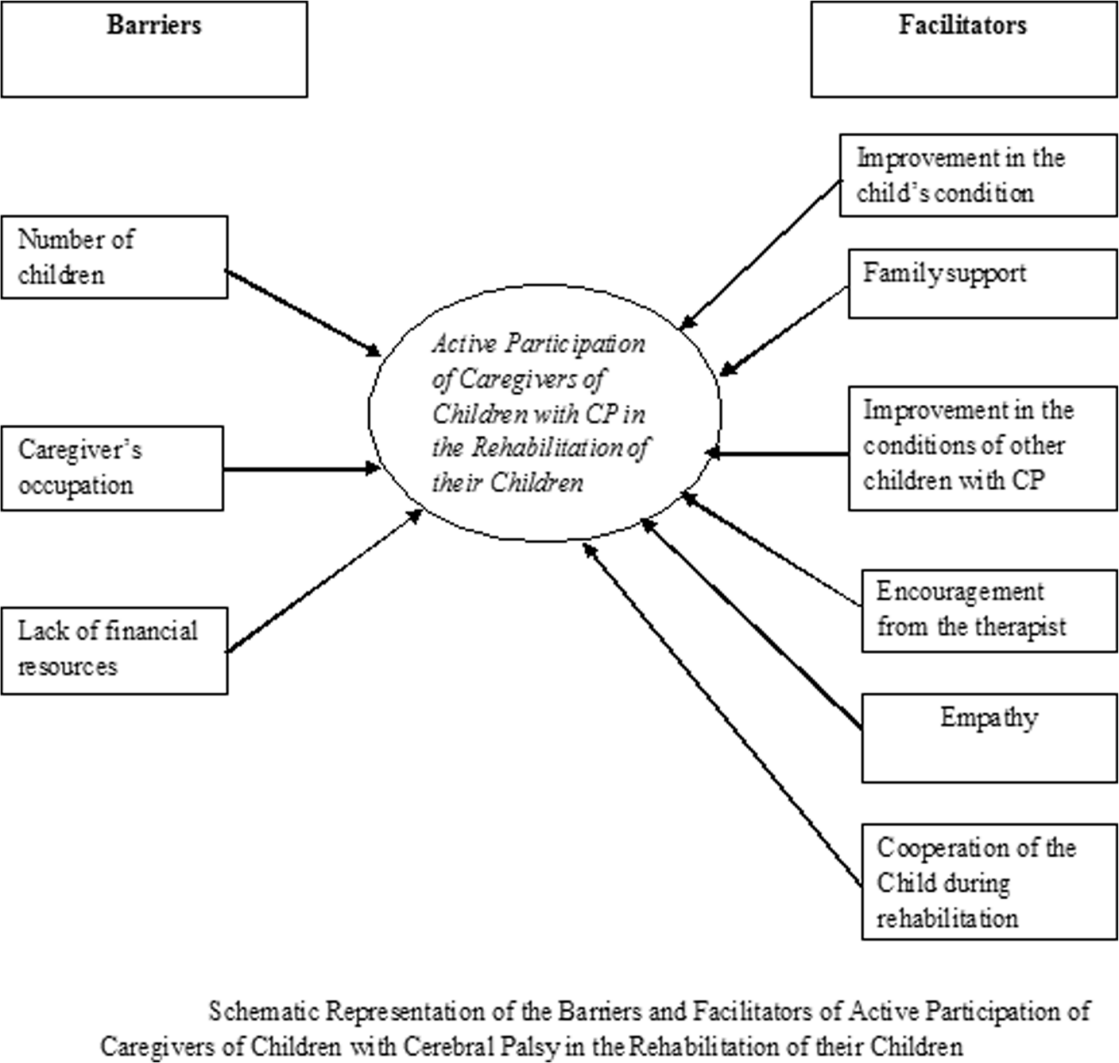
Schematic Representation of the Barriers and Facilitators of Active Participation of Caregivers of Children with Cerebral Palsy in the Rehabilitation of their Children

Following the transcription and coding of the data obtained from the qualitative interview, several themes (minor themes) were generated for both the facilitators and the barriers (major themes). For the facilitators, the themes are improvements in the child’s condition, family support, improvement in the condition of other children with CP, empathy, encouragement by the therapist managing the child and cooperation of the child during home program. The barriers are the number of children the caregiver has, caregiver’s occupation and inadequate financial resources. Figure 1 summarizes the facilitators and the barriers.

### The facilitators

#### Theme 1: improvement in the Child’s condition

What makes the caregivers to bring their children for rehabilitation is hope for help. Therefore, when they observe that their children are getting proper help and are improving, they will be motivated to put more efforts.

*“The time we started attending physiotherapy, my child cannot even sit but now he can stand and even walk a few steps. So this motivates me to carry out the activities that the Physiotherapists asked us to do for him at home”*……………….Participant number 4.

*“It is really encouraging as within the few weeks that my child started receiving Physiotherapy treatment, he can sit for like 3-5 minutes unsupported. He could not sit at the time we first came.”*…………Participant number 13.

*“It is really impressive that within 5 months of treatment my son can now sit and I am confident that by God grace, one day he will walk and even run like his age mates.”*……..Participant number 17.

#### Theme 2: family support

Help from family members can help reduce stress for the caregivers; and make them recharge and prepare to look after their children.

*“Whenever we are asked to use some equipment that will help in the improvement for my child condition, my husband is trying his best to provide such equipments, and whenever he is at home like weekend he used to help me with some home activities like washing clothes. So this is giving me enough time to take care of my child”*……………….Participant number 3.

*“My first daughter is now 18 years old and the second one is 15 years old. Whenever they are at home, they are the ones doing most of the home activities like cooking, and sweeping. So what remains for me is just to take care of my child”*……………….Participant number 17. *“Since the time the Doctor told us that our child is having this condition, my husband employed a girl who is helping me with some work at home. So, this motivates me to utilize my time for taking care of my child”*………………..Participant number 39.

#### Theme 3: improvement in the condition of other children with CP

Seeing other children with CP who have more physical challenges than their child may help caregivers focus on therapy delivery for their children.

*“I have been seeing so many children that their condition is worse than that of my child, but as they are attending clinic, their condition is improving. Thus, I am confident that my child will get better. That is why I am taking my time to do the activities that the therapist asked me to do for him.”*………………Participant number 33.

“*My neighbor has a son that has experienced the same condition with my daughter. But now, he is even attending school. So, that is why I have courage that, with time my daughter too will get to that stage. So that is why I don’t joke with anything the therapist says I should do for her*”………………Participant number 13.

#### Theme 4: encouragement from the therapist managing the child

Encouragements from the therapists are needed in order to boost the morale of the caregivers.

*“Before, my perception was my son will never walk like the other children. But the therapist managing him keep encouraging me that one day my child will even run, and he kept citing many examples to me. So those words of his motivate me to concentrate on my child more than anything*”………………Participant number 35.

#### Theme 5: empathy

*“The rest of my children can perform some of their activities of daily living by themselves, but this child cannot do anything by himself. So this makes me to pity him and pay more attention in taking care of him”*……………Participant number 30.

#### Theme 6: co-operation of the child during the home program

Children who are more cooperative with their rehabilitation make the rehabilitation program delivery easier and more enjoyable for their caregivers.

*“Whenever I am doing the exercises for her at home, she will not be crying as some of the children are doing. So this is encouraging me to do the exercises for her comfortably without difficulties”*……………Participant number 31.

*“My child found most of the exercises enjoyable especially the rollator. So, whenever I am doing the exercise for him he doesn’t even want me to stop. So this is motivating me to do it well for him as he is enjoying it*”…………….Participant number 36.

*“As my child is co-operating during the exercises- nothing like crying, I find it encouraging; and enjoyable to do the exercises for her”*………………Participant number 7.

### The barriers

#### Theme 1: number of children

The number of children a caregiver has, may impact on the kind of attention she may give to the child with CP. In this society, women usually give birth to many children. In this study, the range of number of children is 2 to 8.

*“I have other children in addition to this one and they are all young. Thus, they always need my attention, and this makes me sometimes to delay his home programs”*…………….Participant number 6.

*“As I have told you earlier her mother has died since when she was 3 month; and as you can see me too I have my own baby who is her age mate. But, I have no alternative other than to take her as there is no one to take good care of her other than me as her aunt”*…………….participant number 15.

*“As now she have a younger sister, sometimes I have no choice other than to concentrate on her younger sister especially when she is crying and that makes me to skip some of the activities prescribed for her by the therapist”*……………….Participant number 38.

#### Theme 2: Caregiver’s occupation

Caregiver’s occupation can take some parts of their time, and thus can interfere with the amount of attention they give to the children.

*“I used to sell groundnut and palm oil in my house. So, sometimes customers used to distract my attention from her, but I have no option as the business too is important to our life”*……………….Participant number 5.

*“I am a class room teacher in a primary school. Whenever I am having a class, I used to leave her with a girl that is assisting me in our staff room and sometimes she used to cry before I finish teaching my student”*………………Participant number 34.

“*I am working in secretariat and whenever I left for work, there is a girl that is taking care of her. But I know she cannot do it as appropriately as I am doing”*………………Participant number 31.

#### Theme 3: lack of financial resources

Financial resources are adequately required during rehabilitation especially where payments are made out of pocket. However, in this community, most people live on under a dollar per day [27].

*“Three months ago, the Physiotherapist asked us to buy some equipment that we will be using for him at home (for home program). But up to now, we did not buy some of them because of financial issues; but as soon as we get money we will buy the rest of the equipment”*………………..Participant number 19.

## Discussion

The aim of the study was to explore and identify the barriers and facilitators that caregivers of children with CP encounter to actively participate in the rehabilitation program of their children - from the caregivers’ perspective. The results showed that the facilitators are improvement in the child’s condition, family support, empathy, improvement in the condition of other children with cerebral palsy, encouragement from the therapist managing the child and co-operation of the child during home program. The barriers are number of children the caregiver has, caregiver’s occupation, and lack of financial resources. Therefore, it is important for the therapists to recognize these barriers and facilitators in order to help caregivers to participate actively in the rehabilitation of their children. This is especially that CP is a long-term condition which requires care 24/7. Consequently, the caregivers are considered as those who can help extend the care for their children by actively participating in their rehabilitation [28]. Fortunately, caregivers of children with CP recognize how important physiotherapy is at improving motor and psychosocial functions of their children, and as such they are always willing to carry out the tasks the therapists recommend for their children at home [29]. In addition, when caregivers participated in the rehabilitation of their children, motor function improved better compared to institution based rehabilitation [30]. However, barriers such as number of children the caregiver has, financial constraint and caregiver’s occupation may affect their zeal or willingness to actively participate in the rehabilitation of their children.

Although the number of children a caregiver has can serve as a barrier to their participation in the rehabilitation of their children, a previous quantitative survey had a contrary result. According to Olagunju and colleagues, there was no significant association between number of siblings and compliance with home programs provided by a caregiver to a child with CP [31]. One of the possible reasons for the difference in the findings of the two studies could be because of the different methodologies used. A qualitative approach which was used in the present study has the advantage of getting subtle and nuance insights on a particular phenomenon which cannot be detected by a quantitative study [32]. Secondly, the validity of the questionnaire used in the latter study is uncertain as only poorly explained content and face validation were mentioned. However, other studies reported that number of children a caregiver of a child with CP has can cause so much burden on the caregiver and result in family adjustment [29, 33].

Since the number of children can add more strain and stress to a caregiver, family counseling may be offered to optimize child spacing in order to give adequate attention to the child with CP. This is more so as the caregivers in the present study are still young, and may have potentials to give birth for many years. However, if the siblings are older and they can help in the caregiving, this may facilitate care. In the present study, help or support from the family is a facilitator, and it was previously reported to be a primary source of support for caregivers and that it helps reduce caregiver stress [34, 35]. Therefore, therapists may assist families to identify workload balance whereby one member of the family can do one thing for the child during a particular time, and another some other time. According to Chiluba and Moyo, caregivers of children with CP expressed the need for someone to stand in for them sometimes [33].

It was reported previously that, low income is associated with increased caregiver burden [36, 37]. Additionally, caregivers of children with CP have reported that one of the barriers to accessing care for their children is lack of financial resources [37]. However, CP is a long-term condition, and it requires care in the long-term that may cost huge financial resources. Consequently, cost of care can add more strain to the caregivers and constrain their ability to afford services for their children [38, 39]. This is more difficult in places like Nigeria where people make out of pocket payments for health services. Similarly, in other low resource settings such as in Zambia, families pay healthcare services bills for their children which are most times difficult to afford [33, 38]. Therefore, governments should explore ways to make health services more accessible or subsidize the cost of rehabilitation through enrolling children with CP in National Health Insurance Scheme (NHIS). For now, for instance in Nigeria, the enrollment seems to be restricted to only Government employees and to some extent, students in tertiary institutions who comprise just a small percentage of the population. In contrast, in developed countries such as the United Kingdom, health care services are largely free [40]; and in 2005, out of pocket payment accounted for only 11.9% of total expenditure [41]. Thus, low resource settings such as Nigeria should critically analyze their healthcare delivery and identify economically sustainable systems that will be accessible to all on the basis of need, irrespective of their socioeconomic status.

Another barrier is caregiver’s occupation which can limit the time the caregivers can devote in rehabilitation of their children. Although, there is assistive technology such as Switching that can help decrease caregivers’ effort, energy expenditure and burden [29, 42]; the technology is relatively costly and needs time and skills to operate. Consequently, it is important for therapists to devise other simple and affordable means such as reinforcing family support. Family support or social support can help reduce caregiver stress [37]. This family support can come in form of sharing responsibilities among spouses and members of the household or engaging a paid caregiver if it is possible.

Similarly, improvement in the child’s condition during the rehabilitation program is one of the most important facilitators that motivate the caregivers to put more effort in the rehabilitation of their children with CP. The reasons for this could be because mothers or caregivers of children with CP value and recognize the benefits of Physiotherapy, and they believe that, it is important that the therapy continues [29, 33]. This appreciation is especially as regards to improvement in functional status. Secondly, the family or the caregivers are empowered following CP rehabilitation [43]. However, at the same time the caregivers feel that the care is sometimes not well coordinated and they do not receive the kind of encouragement they feel they need from the therapists [33]. In addition, there is a need for the therapists to incorporate techniques such as motivational interviewing to encourage caregivers to actively participate in the rehabilitation of their children. Motivational interviewing improves self-efficacy [44].

Similarly, even improvement in the conditions of other children with CP other than their own who may have more physical challenges may motivate them to put more efforts in the process of rehabilitation of their children. Number of deficits a child with CP has is associated with increased caregiver burden [34]. Consequently, group therapy, whereby a number of children can have their rehabilitation sessions at the same time may help foster confidence in the caregivers of children with CP. Other facilitators that need to be reinforced by the therapists during rehabilitation include empathy and caregiver encouragement by the therapists. According to Cerebral Palsy Guide, empathy is greatly required in the process of care for a child with CP [45].

However, one of the limitations of this study is that, only participants who were attending Specialists Paediatrics Hospital were included in the study. As such, their views may differ from those attending Primary Healthcare Centers where the system is less advanced than the one in the former. In addition, interview transcripts were not returned to the participants for comments and/ or clarifications. This may affect the quality of the study results.

## Conclusion

Caregiver participation in the rehabilitation of their children with CP is greatly required. However, there are barriers or facilitators that can hinder or encourage their participation. Therefore, therapists need to recognize these barriers and facilitators to help discourage or reinforce them in the caregivers. This can be done through special training of the caregivers and other family members in the principles and practice of care of children with CP and provision of an avenue where caregivers can at any time contact their children’s therapists for help. In addition, government needs to make healthcare services for children with CP accessible and affordable irrespective of the family’s socioeconomic status.

### Implication for policy, research and practice

Although this is a qualitative study representing the perspective of 40 caregivers in the North West of Nigeria, we believe this study contributes new insights to help policymaking in Nigeria. The following implications are suggested:

1. There should a policy on the rehabilitation of children with CP that will make services affordable. This is because, at the moment in Nigeria, the caregivers of children with CP make out of pocket payments for the rehabilitation services for their children. However, in the present study, they expressed lack of financial difficulty as one of the barriers to their active participation in the rehabilitation of their children.
2. The policy should also make services for children with CP accessible. This can be done by having therapists posted to work at the Primary Healthcare Centers since the centers are closer to the people and the communities. However, at the moment in Nigeria, CP is managed in the tertiary, specialized and the general hospitals which are mostly located in the metropolis.
3. There should also be adequate tasks shifting training whereby the caregivers are trained on how to appropriately administer rehabilitation techniques on their children. This is because CPS is a long term condition that requires attention and care 24/7
4. Techniques such as motivational interviewing should be used by the therapists to help encourage caregivers to actively participate in the rehabilitation of their children. This will help improve the self-efficacies of the caregivers to enable them manage the conditions of their children.
5. Research on the rehabilitation of children with CP should focus on innovative ways to help the caregivers have adequate confidence to actively participate in the rehabilitation of their children.

## Data Availability

The data and all materials for this study are available on written request to the corresponding author.

## Abbreviations

ADL: Activities of daily living
CP: Cerebral Palsy
NHIS: National Health Insurance Scheme
